# Supplementary material for: In situ architecture of the algal nuclear pore complex
Source: Nat Commun. 2018 Jun 18;9:2361. doi: 10.1038/s41467-018-04739-y (PMC6006428; doi:10.1038/s41467-018-04739-y)
Supplement: Supplementary file 1 — Supplementary Information [file 41467_2018_4739_MOESM1_ESM.pdf]

SUPPLEMENTARY INFORMATION for Mosalaganti et al.

***In situ* architecture of the algal nuclear pore complex**

Supplementary Figures 1-13
Supplementary Tables 1-2
Supplementary Note 1

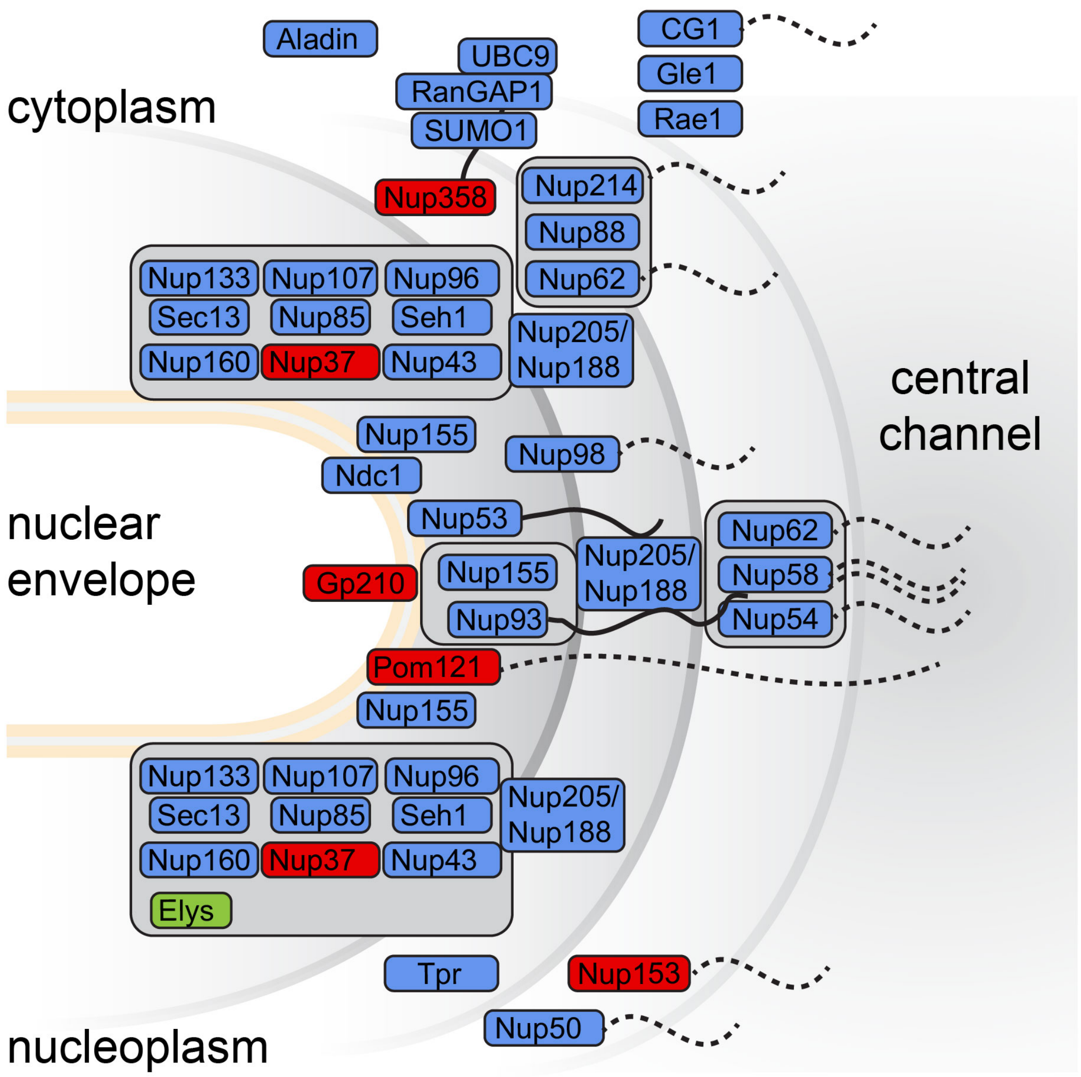

Supplementary Figure 1.

Composition of the *Cr*NPC. Nups identified by searching the *C. reinhardtii* genome are shown in blue and grouped according to subcomplex membership and spatial distribution within the NPC scaffold. Red: absent in *C. reinhardtii*; green: predicted Elys protein lacking a β-propeller domain that is conserved in metazoa.

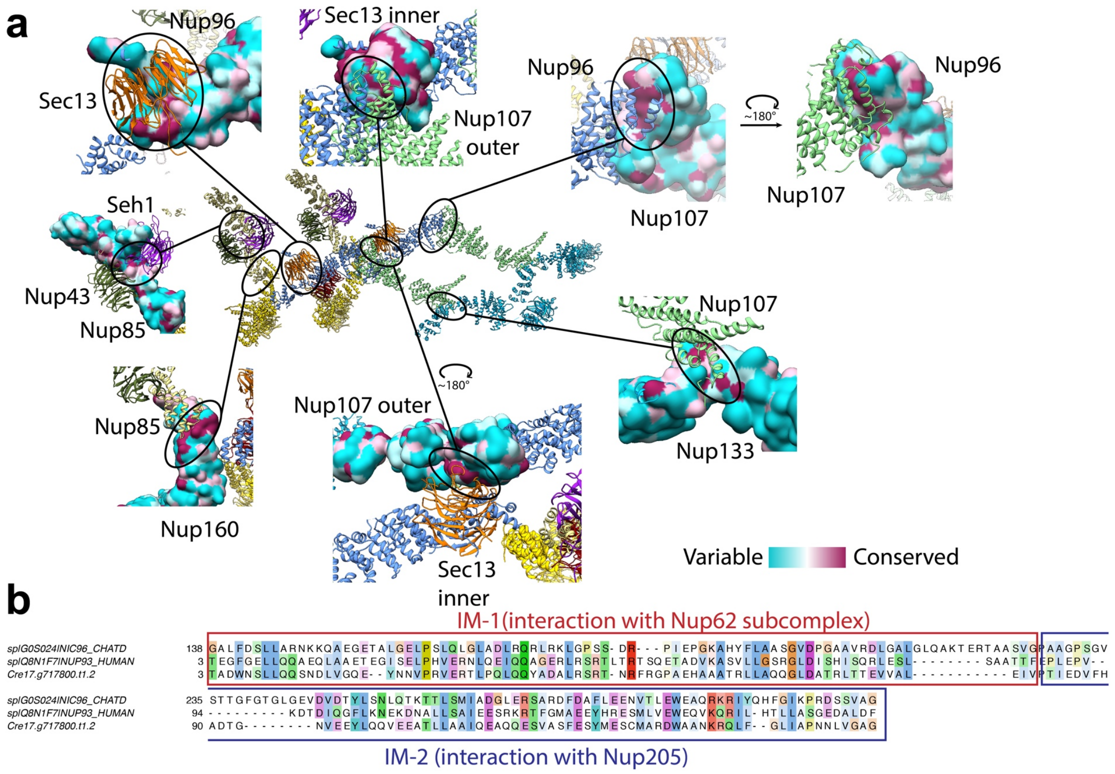

Supplementary Figure 2.

Sequence conservation of inter-subunit contacts in the *Cr*NPC. (a) Conservation of interaction sites within and between Y-complexes. The double arrangement of the human Y-complex is shown in the center. Various *C. reinhardtii* subunits are shown in low resolution surface representation based on corresponding homology models. The surfaces are colored according to sequence conservation between *C. reinhardtii* and human Nups based on pairwise alignments. Conserved residues are red, variable are cyan. Note that conservation could not be evaluated at all interfaces due to the low quality of some regions of predicted *Cr*Nup genes (e.g. the Nup107 C-terminal domain that interfaces with Nup133). **(b)** Sequence alignment between *C. reinhardtii*, human, and yeast for the IM-1 and IM-2 motifs of Nup93, which make contacts to the Nup62 subcomplex and Nup205, respectively. The motifs are conserved.

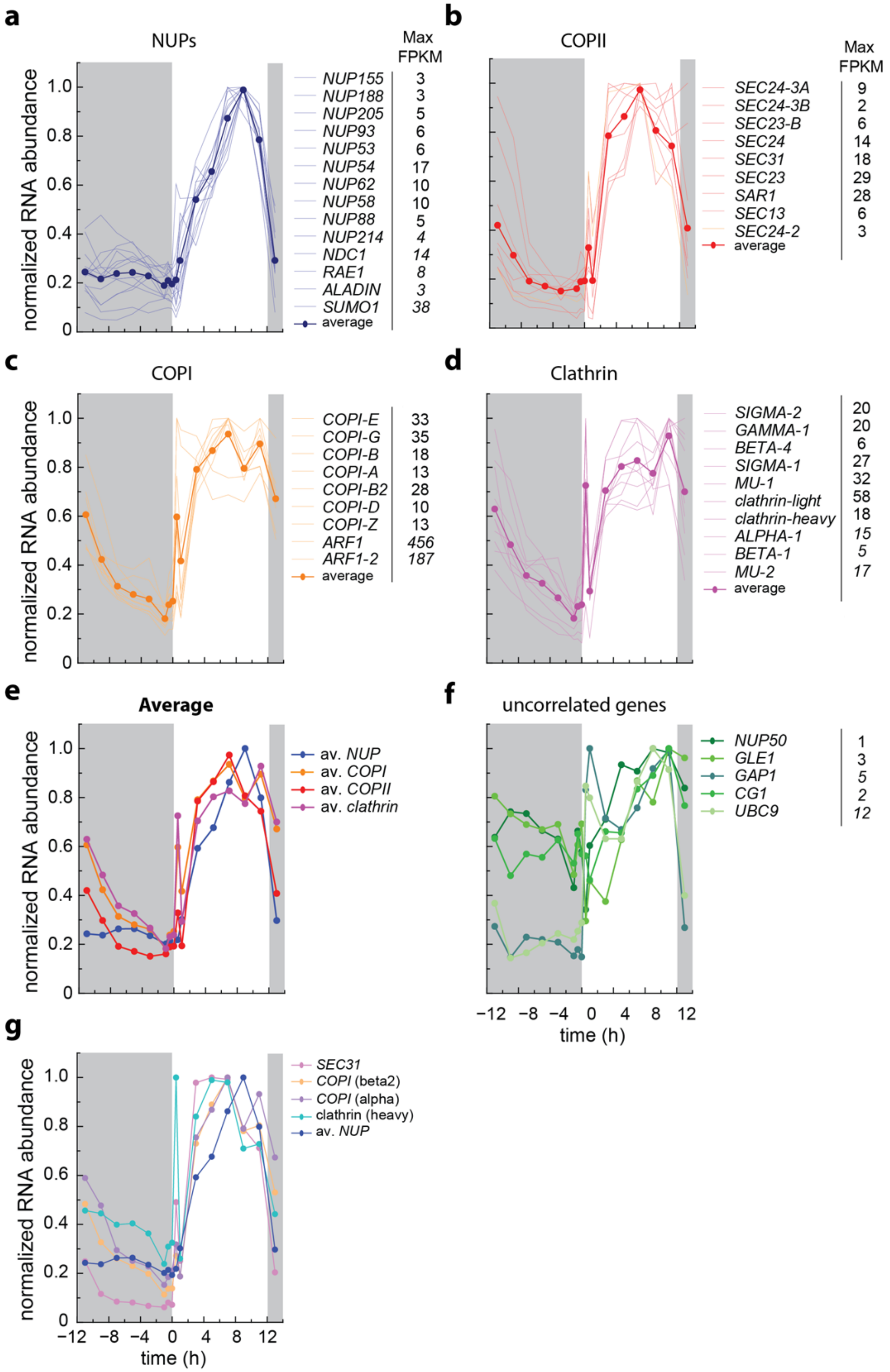
a
NUPs
Max FPKM
NUP155 3
NUP188 3
NUP205 5
NUP93 6
NUP53 6
NUP54 17
NUP62 10
NUP58 10
NUP88 5
NUP214 4
NDC1 14
RAE1 8
ALADIN 3
SUMO1 38
average
b
COPII
Max FPKM
SEC24-3A 9
SEC24-3B 2
SEC23-B 6
SEC24 14
SEC31 18
SEC23 29
SAR1 28
SEC13 6
SEC24-2 3
average
c
COPI
COPI-E 33
COPI-G 35
COPI-B 18
COPI-A 13
COPI-B2 28
COPI-D 10
COPI-Z 13
ARF1 456
ARF1-2 187
average
d
Clathrin
SIGMA-2 20
GAMMA-1 20
BETA-4 6
SIGMA-1 27
MU-1 32
clathrin-light 58
clathrin-heavy 18
ALPHA-1 15
BETA-1 5
MU-2 17
average
e
Average
av. NUP
av. COPI
av. COPII
av. clathrin
f
uncorrelated genes
NUP50 1
GLE1 3
GAP1 5
CG1 2
UBC9 12
g
SEC31
COPI (beta2)
COPI (alpha)
clathrin (heavy)
av. NUP
normalized RNA abundance
0
0.2
0.4
0.6
0.8
1.0
−12 −8 −4 0 4 8 12
time (h)

Supplementary Figure 3.

The expression profiles of *Chlamydomonas* Nup-encoding genes peak together at the end of the day. *C. reinhardtii* cells were grown synchronously in 12 h light (white background) – 12 h dark (grey background) periods so that cells started dividing just once at 11 h during a 24 h period. The dark to light transition occurs at 0 h by convention. RNAs were sequenced at the indicated time points. (**a-e**) The subunits of the NPC, COPII, COPI and clathrin are shown in dark blue, red, orange and magenta, respectively, with the maximum expression set to a value of 1 (see data in Supplementary Data 1, for individual expression estimates in FPKM). (**e**) Overlay of the average expression of individual genes for each complex. The Nup expression pattern is distinct. (**f**) Genes that did not match the expression pattern of any of the complexes investigated. All of these proteins are not part of the NPC scaffold but are more peripherally associated with it and might have additional functions away from the nuclear envelope. Maximum RNA abundances (in FPKM, averaged from three experiments) are shown on the right of each panel. (**g**) Expression patterns of *SEC31* (light magenta), *COPI* beta2 (light orange), *COPI* alpha (light purple), and clathrin heavy chain (cyan) compared to the average NUP expression pattern (blue) as shown in (**a**). These genes are homologous to bona fide Nups but are differentially expressed.

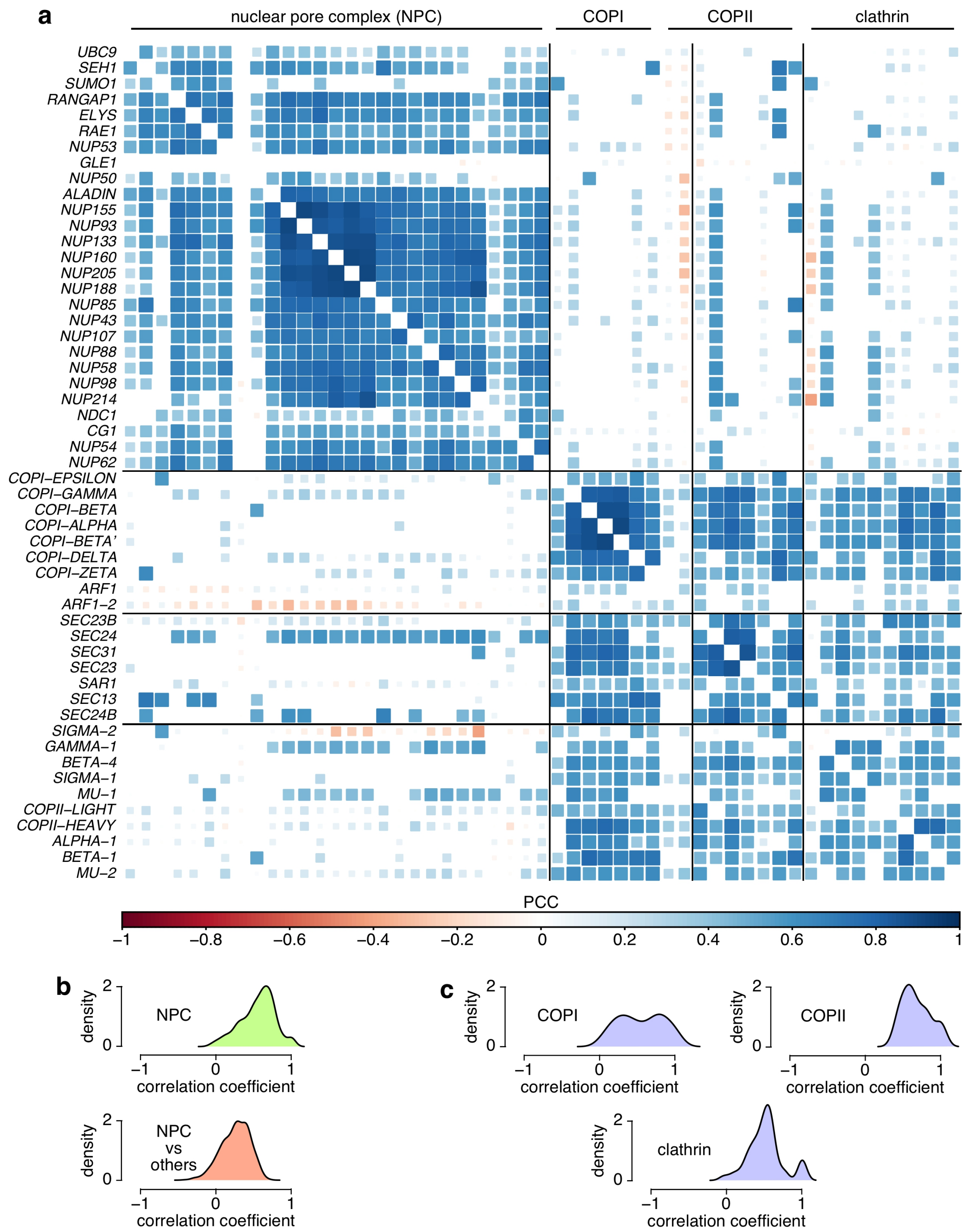

Supplementary Figure 4.

Genes encoding components of the NPC are co-expressed.

(**a**) Correlation matrix of gene expression profiles from genes encoding components of the NPC, COPI, COPII and clathrin protein complexes. Pearson's correlation coefficients (PCCs) were calculated from a dataset derived from 518 RNAseq samples (from 60 independent experiments). Distribution of PCCs for each protein complex: (**b**) NPC (top panel) and NPC vs all other complexes (bottom panel), (**c**) COPI, COPII and clathrin. Nup gene expression is very distinct from the expression of other evolutionary-related membrane coating systems.

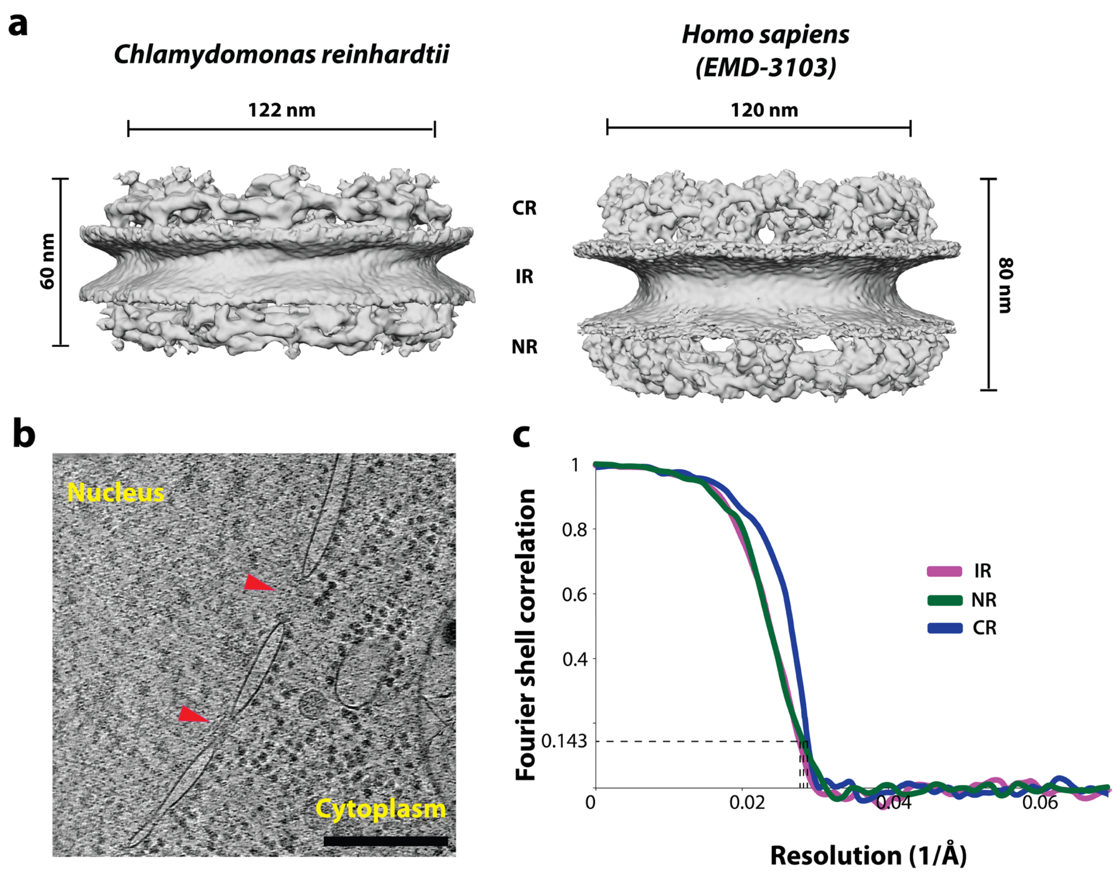

Supplementary Figure 5.

Structure the *Cr*NPC. (a) Side views of the *Cr*NPC (left) and *Hs*NPC (right). **(b)** Slice through an *in situ* tomogram spanning the nucleus and cytoplasm. Red arrowheads indicate NPCs. Scale bar: 200 nm. **(c)** Fourier shell correlation plots for independently averaged inner ring (IR), cytoplasmic ring (CR) and nuclear ring (NR) cryo-EM maps.

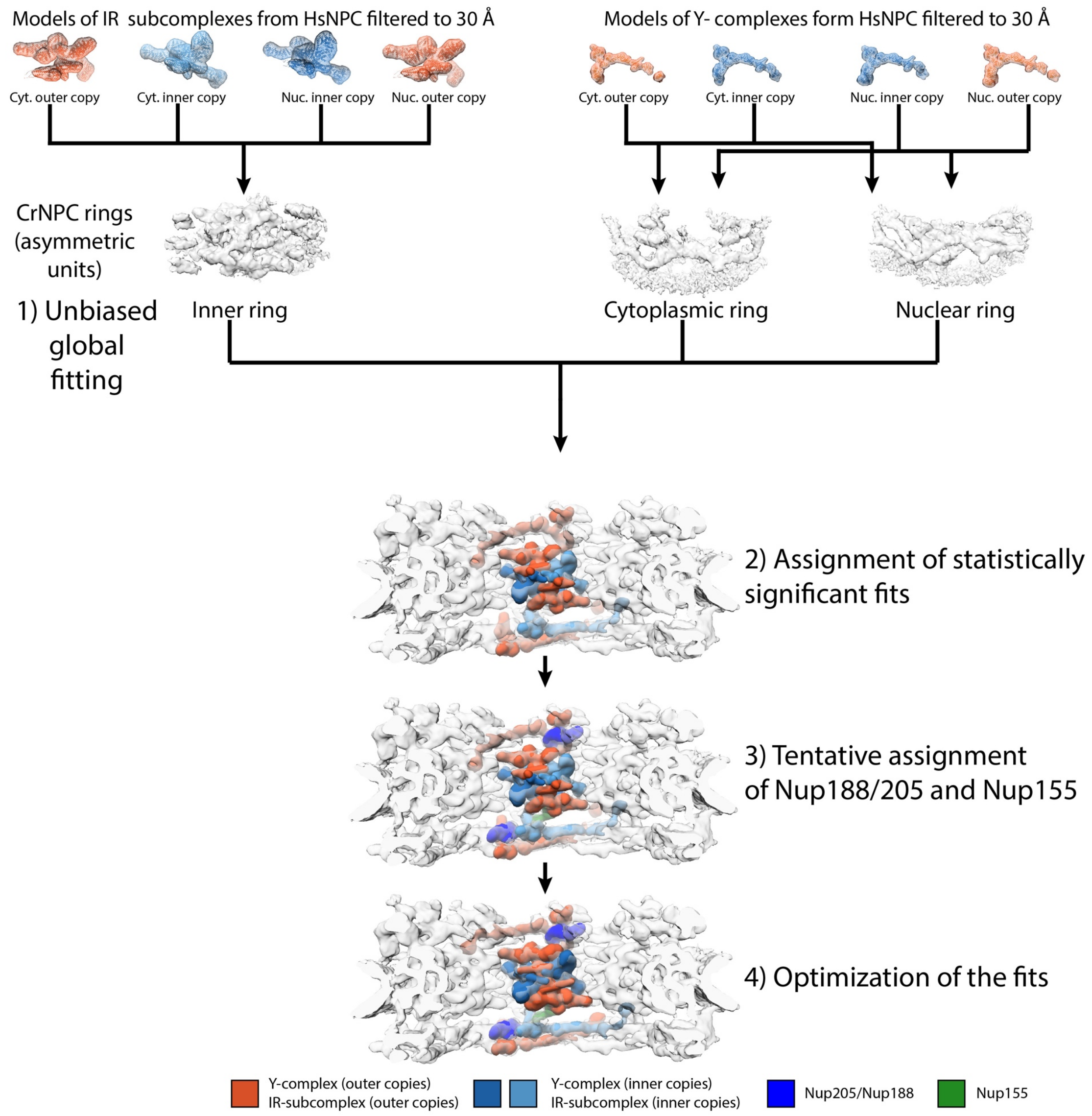

Supplementary Figure 6.

Procedure for assessing the consistency of human subcomplexes with the *Cr*NPC density. The steps are numbered as in Supplementary Note 1, which contains a full description of the procedure. The structural models are shown in their low-resolution representation used for fitting. For step 1, arrows indicate which models were fitted to which part of the map (resulting in 12 fitting runs in total). The *Cr*NPC map is shown either as a weakly filtered single asymmetric unit of cytoplasmic, inner and nuclear rings (step 1) or as a strongly filtered full complex (steps 2-4). The results of the individual fitting runs are presented in Supplementary Figures 7-8. Abbreviations: cytoplasmic ring (CR), inner ring (IR), nuclear ring (NR), cytoplasmic face (cyt.), nuclear face (nuc.).

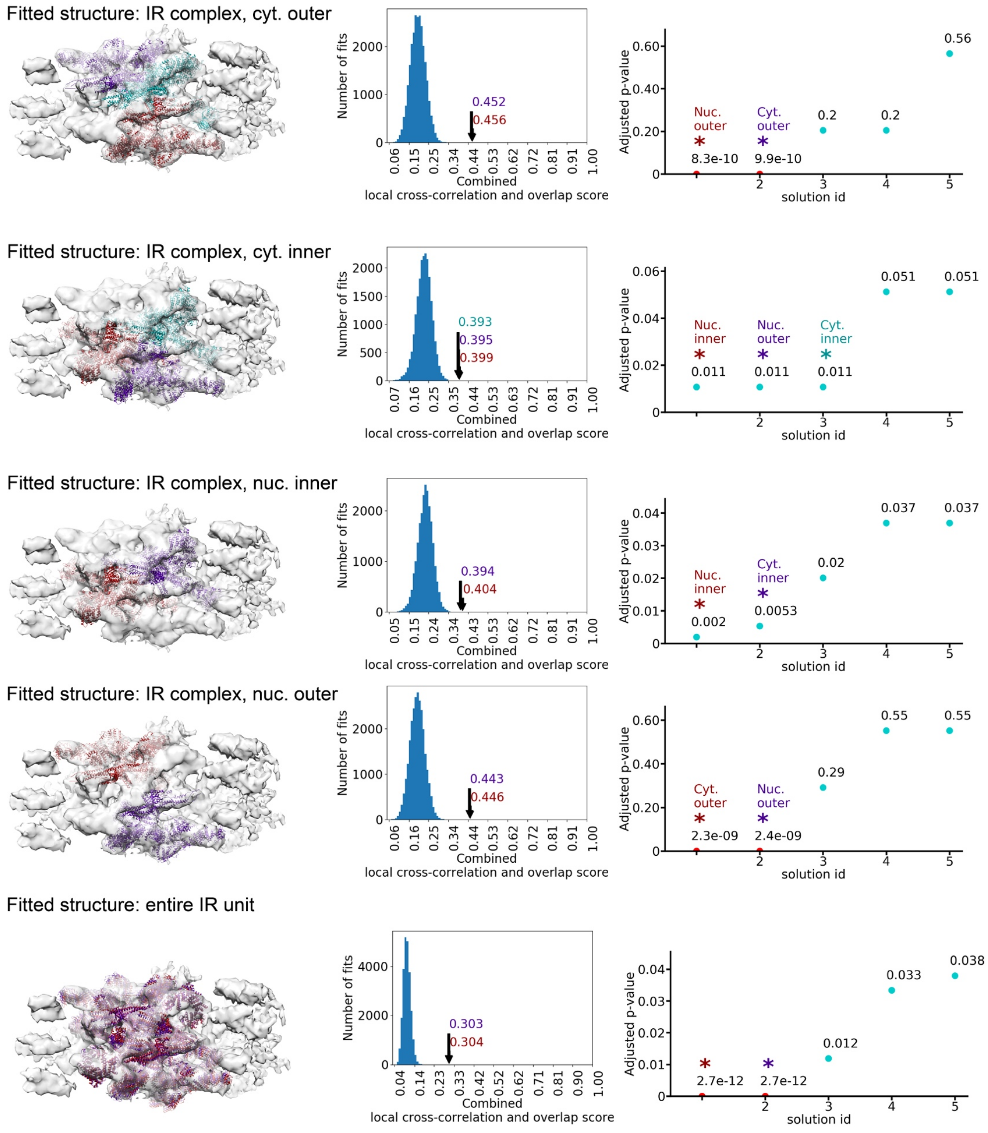

Supplementary Figure 7.

Systematic fitting of inner ring (IR) components into the *Cr*NPC map. The four IR protomers of the *Hs*NPC fitted independently as rigid bodies by unbiased global search using UCSF Chimera. Each protomer consists of Nup155, Nup93, Nup62, Nup54, Nup58 and Nup188/Nup205. Moreover, the entire asymmetric unit of the human IR was fitted as a rigid body. All models were fitted into the *Cr*NPC map by unbiased global search using UCSF Chimera and scored as explained in Supplementary Note 1. Each row shows a plot of the top five *p* values (*right*), the histogram of raw scores (*middle*) and the visualization of the most significant fits (*left*). The fits representing the arrangement equivalent to *Hs*NPC are marked with an asterisk and colored red, magenta, and cyan. The positions within the IR unit are labelled over the asterisks, except when the entire IR unit is fitted. The two significant fits for the entire IR unit correspond to equivalent fits rotated around the approximate C2 symmetry axis of the IR. The structural models are shown in atomic representation but were filtered to 30 Å for the purpose of fitting. Abbreviations: inner ring (IR), cytoplasmic face (cyt.), nuclear face (nuc.).

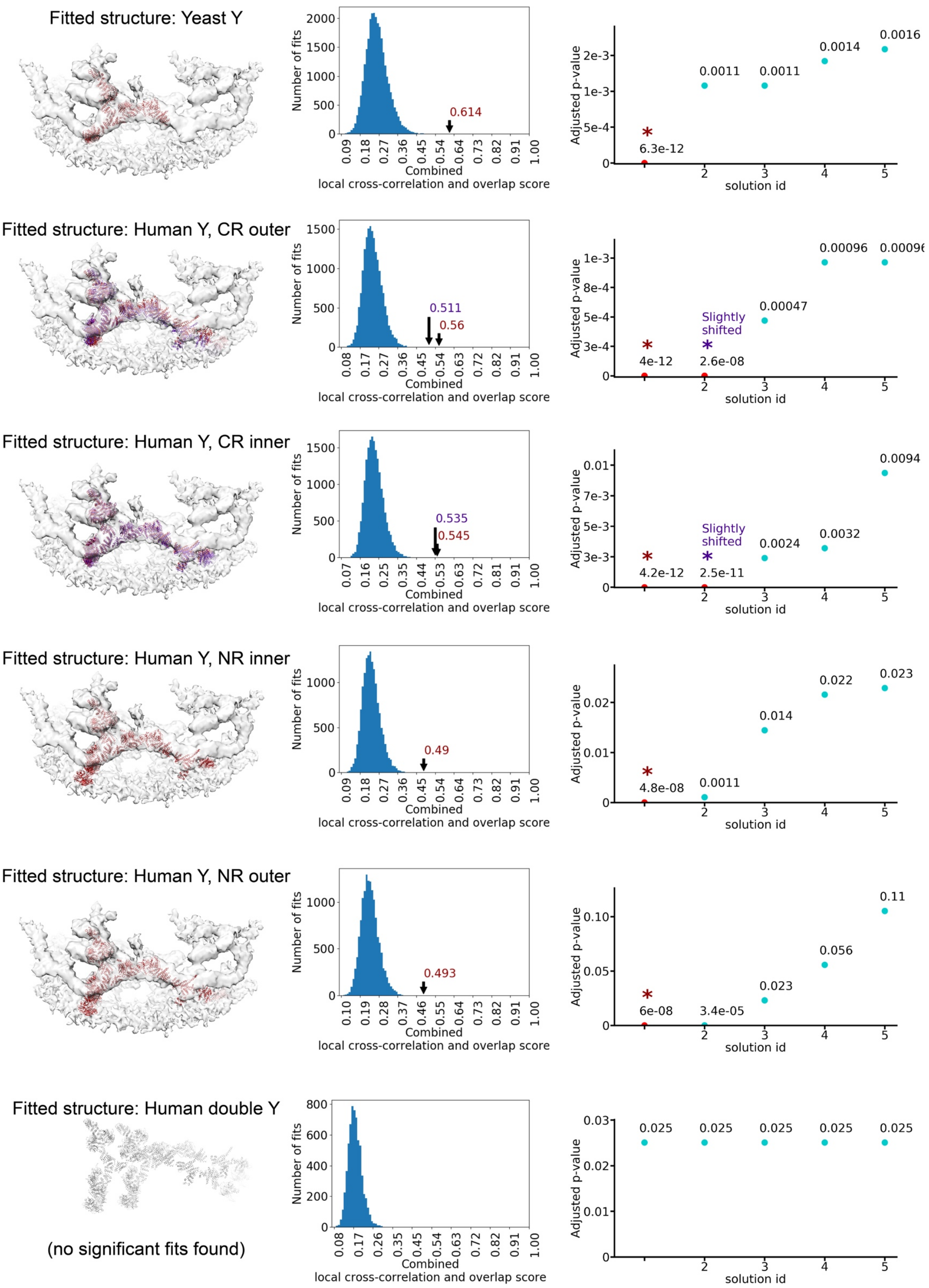

Supplementary Figure 8.

Systematic fitting of the Y-complex into the cytoplasmic ring (CR) of the *Cr*NPC map. The crystal structure of the yeast Y-complex (PDB ID: 4XMM) and models of the human Y-complex fitted independently as rigid bodies by unbiased global search and scored as explained in the Supplementary Note 1. Each row shows a plot of the top five *p* values (right), the histogram of raw scores (middle) and the visualization of the most significant fits (left). The fits representing the arrangement equivalent to *Hs*NPC are marked with an asterisk and colored red and magenta. The "slightly shifted" fits indicate fits that are very similar to the top fit but with some minor differences in the orientation. The models of the Y-complexes are shown in atomic representation but were filtered to 30 Å for the purpose of fitting. Abbreviations: cytoplasmic ring (CR), nuclear ring (NR).

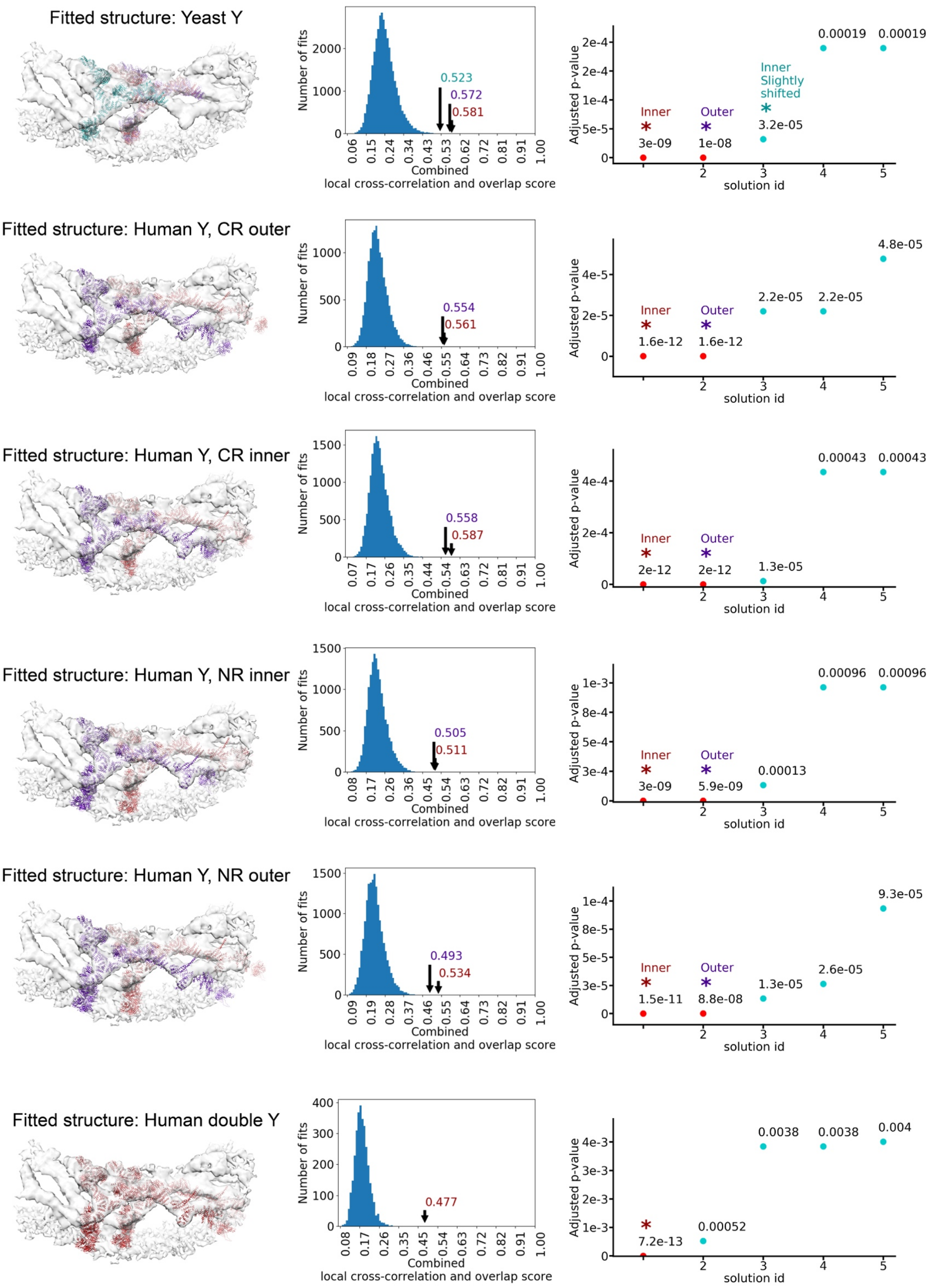

Supplementary Figure 9.

Systematic fitting of the Y-complex into the nuclear ring (NR) of the *Cr*NPC map. The crystal structure of the yeast Y-complex (PDB ID: 4XMM) and models of the human Y-complex fitted independently as rigid bodies by unbiased global search and scored as explained in the Supplementary Note 1. Each row shows a plot of the top five *p* values (right), the histogram of raw scores (middle) and the visualization of the most significant fits (left). The fits representing the arrangement equivalent to *Hs*NPC are marked with an asterisk and colored red, magenta, and cyan. The “slightly shifted” fits indicate fits very that are similar to the top fit but with some minor differences in the orientation. The models of the Y-complexes are shown in atomic representation but were filtered to 30 Å for the purpose of fitting. Abbreviations: cytoplasmic ring (CR), nuclear ring (NR).

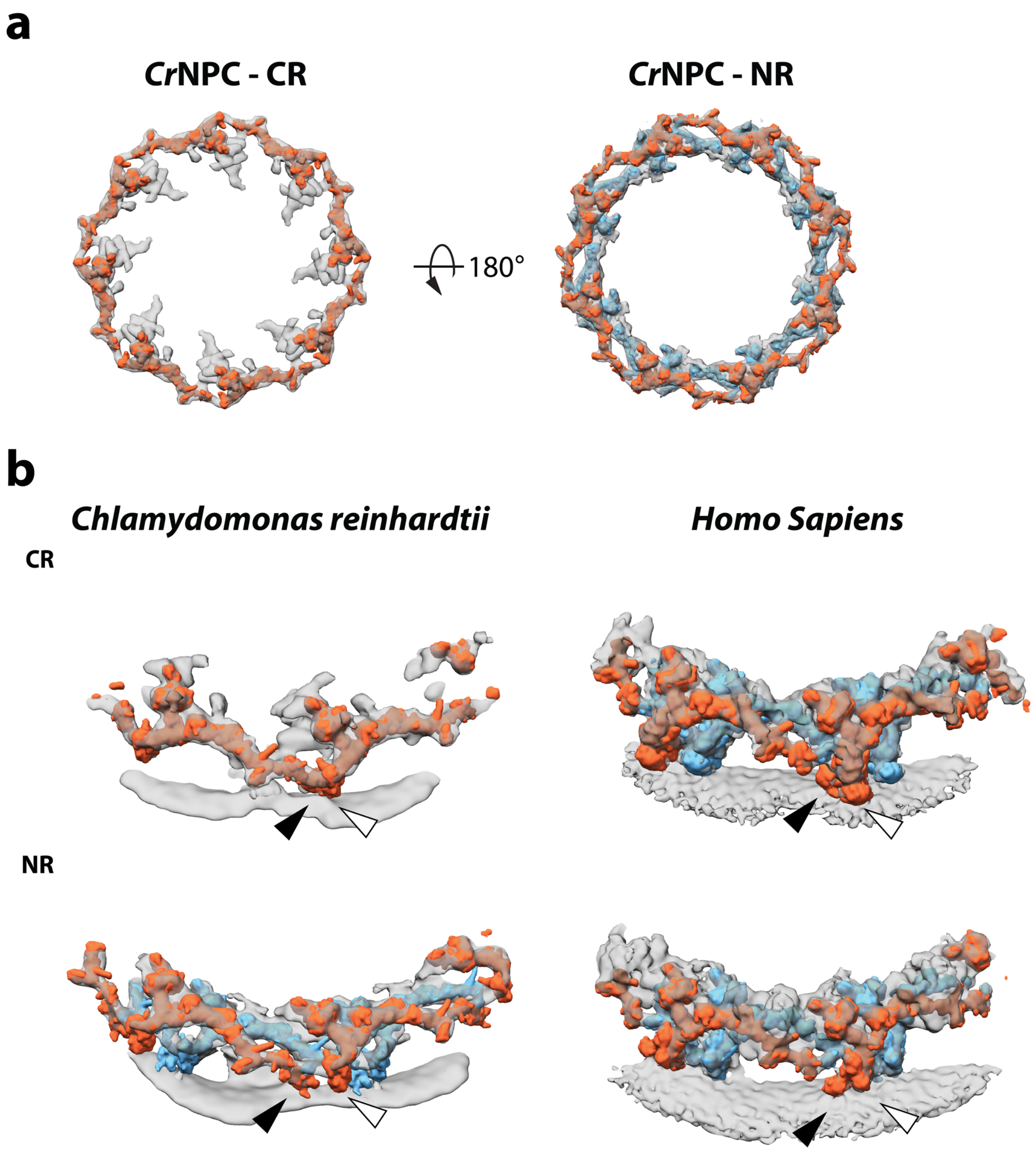

Supplementary Figure 10.
Head-to-tail interaction of the Y-complexes. (a) Cytoplasmic and nuclear face views of the *Cr*NPC with eight (orange) and sixteen (orange: outer; blue: inner) Y-complexes fit respectively, highlighting how the Y-complexes make head-to-tail interactions in a clockwise manner. **(b)** The interface between two asymmetric subunits of the *Cr*NPC (left) and *Hs*NPC (right), viewed either from the cytoplasmic (CR) or the nucleoplasmic (NR) side. Arrowheads (white: head, black: tail) indicate head-to-tail Y-complex interactions, which are similar for both species.

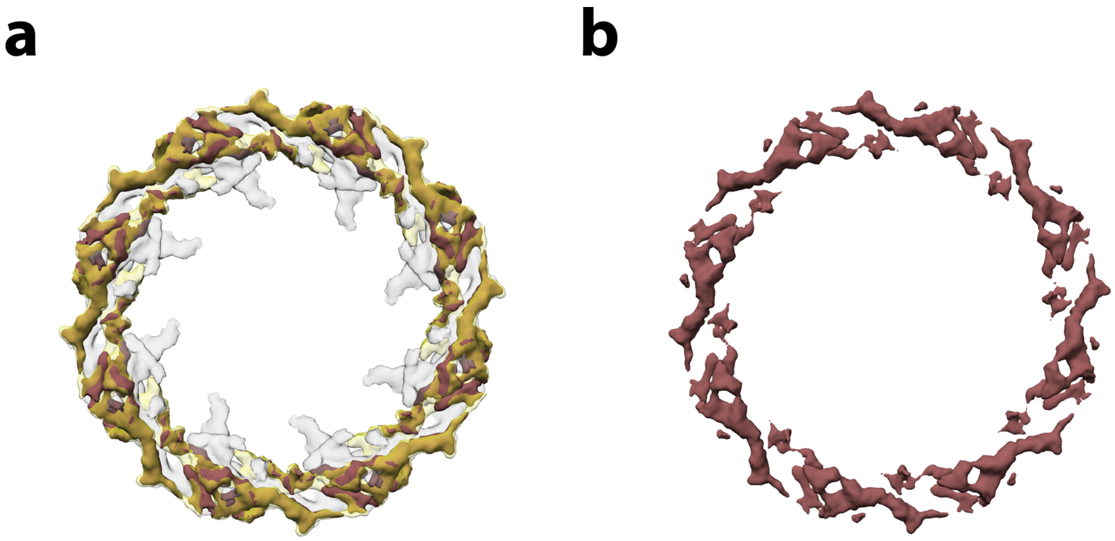

Supplementary Figure 11.
***Cr*NPC outer ring difference map. (a)** The *Cr*NPC cytoplasmic ring density (gray) was subtracted from the *Cr*NPC nuclear ring density (yellow) to reveal the additional cryo-EM density specific to the nuclear ring (brick red). **(b)** This difference density clearly shows eight additional Y-complexes (brick red).

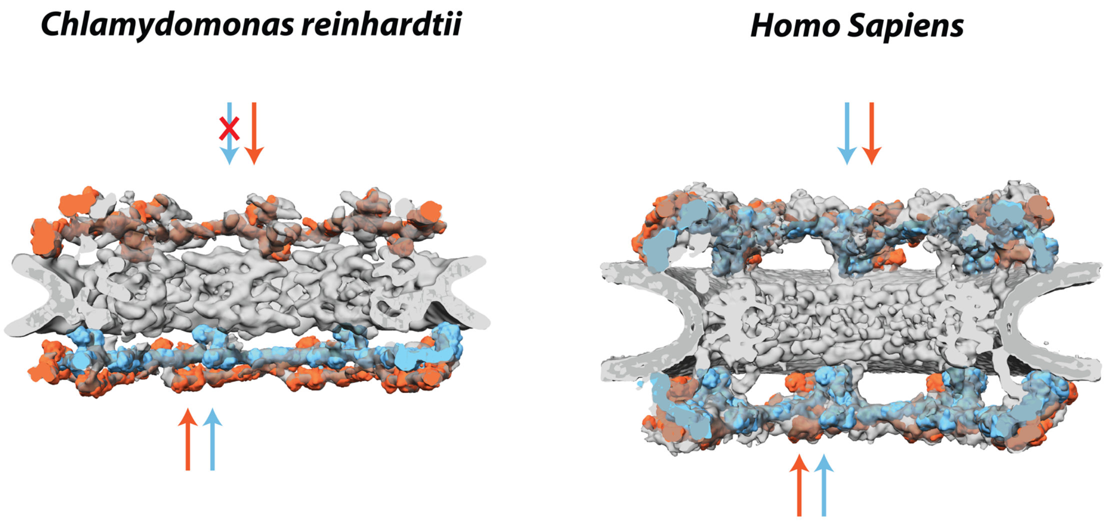

Supplementary Figure 12.
Rotational registration of the Y-complexes. The inner copies of both the nuclear and cytoplasmic Y-complexes in the *Hs*NPC (light blue, right) align where Nup160 contacts the inner nuclear membrane (blue arrows). In contrast, the membrane contacts of the outer copies (orange arrows) are farther apart. Y-complexes in the *Cr*NPC (left) are arranged in a highly similar rotational register, with the exception of the missing inner copy in the cytoplasmic ring (red cross).

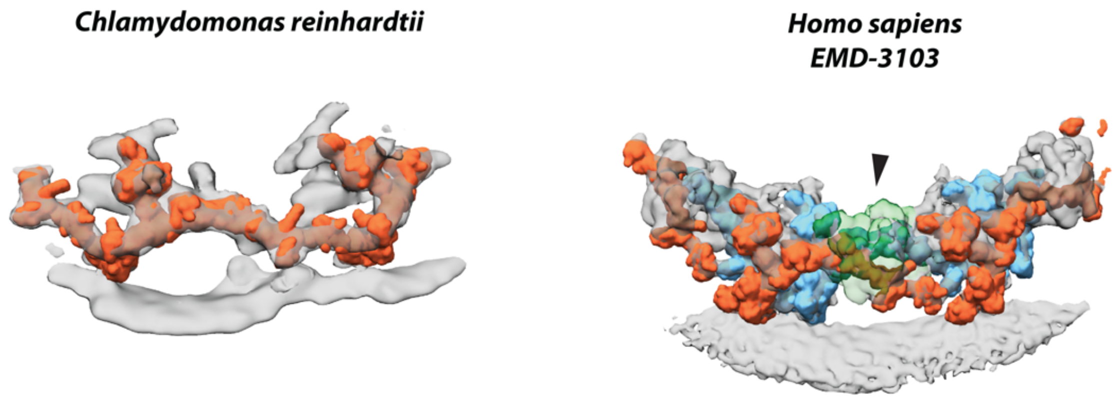

Supplementary Figure 13.
Comparison between the cytoplasmic rings of the *Cr*NPC and *Hs*NPC. Partial segments of the rings are shown. Nup358 (green density, right panel) is a protein that localizes to cytoplasmic side of the *Hs*NPC and holds the inner and outer Y-complexes together. The *Cr*NPC cytoplasmic ring (left) lacks Nup358 and, correspondingly, has only one copy of the Y-complex per asymmetric unit.

Supplementary Table 1.

Accession numbers from the Phytozome platform[1] for Nups identified in *C. reinhardtii*.

Nucleoporin	Transcript ID	Comments
**Nup160**	Cre07.g324000.t1.2	Complete sequence but several low complexity insertions comparing to human Nup160 (likely gene prediction errors).
**Nup133**	Cre12.g521750.t1.2	Several low complexity insertions comparing to human Nup133 (likely gene prediction errors).
**Nup107**	Cre10.g425675.t1.1 (NTER) Cre10.g425700.t1.2 (CTER)	Two separate entries in for NTER and CTER, but close to each other on the chromosome - likely gene prediction error and this is the same gene, as RNAseq reads bridge both genes, indicating a single, larger locus.
**Nup85**	Cre08.g383650.t1.2	Complete sequence but several low complexity insertions comparing to human Nup85 (likely gene prediction errors).
**Seh1**	Cre03.g192000.t1.2	
**Nup43**	Cre02.g090600.t1.2	
**Nup98/96**	Cre02.g086887.t1.1	Some low complexity insertions comparing to human Nup98/96 (likely gene prediction errors).
**Sec13**	Cre17.g715750.t1.2	
**Nup37**	Not identified	
**Elys**	Cre16.g683437.t1.1	Does not contain the N-terminal β-propeller domain present in human Elys.
**Nup153**	Not identified	
**Nup1 (plant)**	Not identified	
**Nup358**	Not identified	
**Tpr**	Cre11.g468356.t2.1	Some low complexity insertions comparing to human Tpr (likely gene prediction errors). (There is an alternative transcript of same gene, Cre11.g468356.t1.1, but it has a one more erroneous exon comparing to human Tpr, not fully supported by RNAseq reads).
**Nup155**	Cre09.g394350.t1.2	NCBI and Uniprot sequence identical to each other, but not to Phytozome. Phytozome sequence seems to have more regions aligning to hNup155, but also has low complexity insertions.
**Nup188**	Cre13.g587800.t1.1	Phytozome has best coverage. Several long wrong regions and likely missing regions
**Nup93**	Cre17.g717800.t1.2	NCBI and Uniprot sequence identical to each other, but not to Phytozome. Phytozome sequence has more regions aligning to hNup93, but also has low complexity insertions.
**Nup205**	Cre13.g578100.t1.1	NCBI and Uniprot sequence identical to each other, but not to Phytozome. Phytozome sequence seems to have more regions aligning to hNup205, but also has low complexity insertions.
**Nup53**	Cre12.g553050.t1.2	Almost entire crNup53 sequence aligns to atNup53 (with some bigger deletions).
**Nup54**	Cre12.g542950.t1.1	Complete sequence, no unexpected low-complexity insertion in the coiled-coil domain.
**Nup62**	Cre16.g677700.t1.1	Complete sequence, no unexpected low-complexity insertion in the coiled-coil domain.
**Nup58**	Cre16.g685217.t1.1	Complete sequence, one small low-complexity insertion (likely gene prediction error).
**Nup88**	Cre06.g278900.t1.1	Complete sequence, several low-complexity insertions (likely gene prediction errors).
**Nup214**	Cre07.g313500.t1.2	Complete sequence, several low-complexity insertions (likely gene prediction errors).
**Rae1**	Cre06.g278097.t1.1	Complete sequence.

**Nup50**	Cre15.g641000.t2.1	Complete sequence.
**Ndc1**	Cre03.g197550.t1.2	Includes both the transmembrane and C-terminal domain.
**Gp210**	Not identified	
**Aladin**	Cre10.g466300.t1.2	
**Gle1**	Cre06.g300900.t1.1	
**RanGAP1**	Cre11.g479250.t1.2	
**CG1**	Cre06.g254700.t1.1	
**SUMO1**	Cre16.g675637.t1.1	There are three other very similar SUMO-like proteins (Cre16.g675861.t1.1, Cre16.g675749.t1.1) and because of high mutual sequence similarity, it is non trivial to determine which of the four genes exactly corresponds to the human SUMO1. Only Cre16.g675637 is co-expressed with Nups.
**UBC9**	Cre02.g142000.t1.2	Shows positive co-expression with NUPs.

Supplementary Table 2.

Cryo-EM data collection statistics for *Chlamydomonas reinhardtii* nuclear pore complex (EMDB-4355)

**Data collection and processing**	
Magnification	42000x
Voltage (kV)	300
Electron exposure (e–/Å^2)	~100
Defocus range (μm)	-4 to -5.5
Pixel size (Å)	3.42
Tilt angle range	-60^0 to +60^0
Tilt increment	2^0
Symmetry imposed	C8
Initial Tomograms (no.)	136
Initial sub-tomograms (no.)	78
Final sub-tomograms (no.)	78
Map resolution (Å)<br>$FSC_{0.143}$ threshold	30
**Refinement**	N/A

Supplementary Note 1.

Assignment of subcomplexes within the *Cr*NPC map

To assign densities of the *Cr*NPC map to specific subcomplexes, a hierarchical fitting procedure (Supplementary Fig. 6) was applied.

i) Unbiased global fitting. An unbiased global fitting approach was performed using structural models of various human subcomplexes derived from previously published structures[2,3]. The Y-complex was complemented with an additional Nup96 C-terminal region model based on the yeast Y-complex crystal structure (PDB ID: 4XMM)[4]. Because of the lower resolution of the *Cr*NPC map, all models were low-pass filtered to 30 Å. The resulting model maps were then independently fitted into the *Cr*NPC cryo-EM density using global fitting as implemented in UCSF Chimera[5]. The fitting was performed independently for cytoplasmic, inner and nuclear rings. The regions of the nuclear envelope distant from apparent contact points between membrane and protein densities were erased prior to the fitting to eliminate fits significantly overlapping with the membrane. All fitting runs were performed using 1,000,000 random initial placements, local cross-correlation (Chimera's correlation about the mean) as a fitting metric, and the requirement of at least 30% of the model map to be covered by the *Cr*NPC density envelope defined at low threshold. For each fitted model, this yielded between 14,000-37,000 fits after clustering.

ii) Assignment of statistically significant fits. For each fitting run, the statistical significance of the fits was assessed as a p-value calculated from the normalized cross-correlation scores. To calculate the p-values, the cross-correlation scores were first transformed to z-scores (Fisher's z-transform) and centered, from which two-sided p-values were computed using standard deviation derived from an empirical null distribution (derived from all obtained fits and fitted using the fdrtool R-package)[6]. All p-values were corrected for multiple testing using the Benjamini-Hochberg procedure. However, when using the standard local cross-correlation as a metric, very few significant fits were identified and only for the inner ring. An inspection of the top fits revealed that fits similar to the *Hs*NPC arrangement were found among top fits, but the empirical null distribution of all fits (used for p-value calculation) contained many fits with high cross-correlation values but low overlap with the *Cr*NPC density, which consequentially reduced the p-values of top fits. Therefore, to obtain an appropriate empirical null distribution, we used a new scoring metric based on the combination of a local cross-correlation score and overlap with the density[7]. The resulting procedure resulted in re-ranking of the fits, and several fits clearly separated from the null distribution with significant p-values (Supplementary Figures 7-9).

All non-redundant statistically significant fits were placed in the model, leading to assignment of four copies of the inner ring subcomplexes, two copies of the Y-complex in the nuclear ring, and one copy of the Y-complex in the cytoplasmic ring. These three fits reproduced the arrangement observed in the *Hs*NPC, reinforcing the confidence in the fits.

iii) Tentative assignment of Nup188/205 and the Nup155 connector. Analysis of the difference density between the resulting model and the *Cr*NPC map revealed characteristic unassigned densities on the nuclear side of the *Cr*NPC that matched the positions of Nup188/205 and the connector Nup155 in the *Hs*NPC. Based on both the shape and positional similarity, these densities were tentatively assigned as Nup188/205 and Nup155. Because the shapes of the Nup188 and Nup205 crystal structures are similar at 30 Å resolution, the densities could not be unambiguously assigned to one of the two Nups.

iv) Optimization of the fits. Visual inspection of the fits indicated conformational differences between the fitted human subcomplexes and *Cr*NPC densities, especially in the stem region of the Y-complex. Therefore, the fits were optimized by local re-fitting of individual subunits or domains. The adjustments of the fits were limited to splitting the structures at the hinges between domains and local fitting of the domains. The local fitting always kept the fits close to the initial rigid body fit of the entire subcomplex. It must be noted that due to the lower resolution of the *Cr*NPC map, the final fits should not be interpreted at atomic resolution; the flexible fitting merely aids in the assignment of densities and segmentations. Finally, Nup37 and the Elys β-propeller, which lacked corresponding densities in the *Cr*NPC map and were not identified in the *C. reinhardtii* genome, were removed from the Y-complex fits.

In addition to the above procedure, several validation runs were performed using the entire inner ring asymmetric unit (which led to a statistically significant hit, Supplementary Fig.7) and models with a double Y-complex arrangement as in the *Hs*NPC (which led to statistically significant fit only for the nuclear ring, Supplementary Figs. 8-9).

Supplementary References

1 Goodstein, D. M. *et al.* Phytozome: a comparative platform for green plant genomics. *Nucleic Acids Res* **40**, D1178-1186 (2012).
2 von Appen, A. *et al.* In situ structural analysis of the human nuclear pore complex. *Nature* **526**, 140-143 (2015).
3 Kosinski, J. *et al.* Molecular architecture of the inner ring scaffold of the human nuclear pore complex. *Science* **352**, 363-365 (2016).
4 Stuwe, T. *et al.* Nuclear pores. Architecture of the nuclear pore complex coat. *Science* **347**, 1148-1152 (2015).
5 Pettersen, E. F. *et al.* UCSF Chimera--a visualization system for exploratory research and analysis. *J Comput Chem* **25**, 1605-1612 (2004).
6 Strimmer, K. fdrtool: a versatile R package for estimating local and tail area-based false discovery rates. *Bioinformatics* **24**, 1461-1462 (2008).
7 Joseph, A. P., Lagerstedt, I., Patwardhan, A., Topf, M. & Winn, M. Improved metrics for comparing structures of macromolecular assemblies determined by 3D electron-microscopy. *Journal of structural biology* **199**, 12-26 (2017).
