# Supplementary material for: In situ architecture of the algal nuclear pore complex
Source: Nat Commun. 2018 Jun 18;9:2361. doi: 10.1038/s41467-018-04739-y (PMC6006428; doi:10.1038/s41467-018-04739-y)
Supplement: Supplementary file 3 — Description of Additional Supplementary Files [file 41467_2018_4739_MOESM3_ESM.pdf]

Description of Additional Supplementary Files

File Name: Supplementary Movie 1

Description: Fit of the 24 Y-complexes (outer: orange, inner: light blue) into the CrNPC cryo-EM density map. Accompanies Fig. 3a.

File Name: Supplementary Data 1

Description: Individual expression estimates of the components of NPC, COP I, COP II and Clathrin, in Fragments Per Kilobase of transcripts per million mapped reads (FPKM).
